# Genomic Sequencing Insights Into a Rare Case of High-Risk Diffuse Large B-cell Lymphoma (DLBCL) Associated With Rubinstein-Taybi Syndrome

**DOI:** 10.7759/cureus.91682

**Published:** 2025-09-05

**Authors:** Sreeja Ponnam, Prathima Guntipalli, Supriya Koya

**Affiliations:** 1 Medicine, University of Missouri–Kansas City (UMKC) School of Medicine, Kansas City, USA; 2 Preventive Medicine, Larkin Community Hospital, Miami, USA; 3 Hematology and Oncology, OU Health Stephenson Cancer Center, Oklahoma City, USA

**Keywords:** braf (v-raf murine sarcoma viral oncogene homolog b1), crebbp (cyclic-amp-regulated enhancer-binding protein), diffuse large b-cell lymphoma (dlbcl), double hit lymphoma, immunostaining, myc (avian myelocytomatosis viral oncogene homolog), r-epoch, rubinstein-taybi syndrome (rsts)

## Abstract

We report a rare case of a Rubinstein-Taybi syndrome (RSTS) patient who developed diffuse large B-cell lymphoma (DLBCL). RSTS is a very rare, genetic, developmental disease. RSTS patients have been reported to have some predilection for certain cancers. The patient had both germline and somatic mutations in the *CREBBP* (cyclic-AMP-regulated enhancer-binding protein) gene. *CREBBP* has been reported to be associated with poor prognosis and less response to chemotherapy. We treated the patient with Rituxan with EPOCH (R-EPOCH) with excellent response. Based on the rarity of this diagnosis and the development of lymphoma in this patient, we sought to establish a genomic connection between RSTS and its association with high-risk DLBCL. The distinct response observed in this case warrants further investigation into the potential benefits of intensified therapies in DLBCL patients harboring specific genetic alterations such as *CREBBP*.

## Introduction

First described in 1963, Rubinstein-Taybi syndrome (RSTS) is a rare, genetic, developmental disease [1,2]. The incidence of RSTS is one in 100,000 to 125,000 live births for RSTS. No accurate diagnostic criteria are available. However, RSTS is characterized by the slow development of height and weight, microcephaly, dysmorphic facial features, intellectual disability, broad thumbs, and big first toes [2]. Other skeletal abnormalities and complicated neuroradiological issues are also seen, along with a typical presentation of an enlarged first finger and clinodactyly of the fifth finger [2]. Certain cancers, specifically those with neural and developmental origins, such as neuroblastoma, medulloblastoma [3,4], and certain lymphomas and leukemias, have also been reported in RSTS patients [5].

Previous studies reported that the hybridization signal was present on only one allele on 16p13.3, leading to RSTS [6,7]. Later, studies in RSTS patients discovered mutations in the gene encoding the cyclic-AMP-regulated enhancer-binding protein (*CREBBP*) in 16p13.3 [8] and alterations in the E1A-binding protein p300 (*EP300*) [2]. More than half of the RSTS patients had reported mutations of the *CREBBP* gene [9]. The *CREBBP* gene and its homolog, *EP300* on chromosome 22, are involved in several cellular activities as transcriptional co-activators in varying signaling pathways, such as DNA repair, growth, differentiation, apoptosis of cells, and tumor suppression [10]. Mutations in *CREBBP* and *EP300* genes were identified by molecular analysis, where 50-70% of RSTS patients were identified with pathogenic variants of the *CREBBP* gene and 5-8% of RSTS patients reported mutations in the *EP300* gene [2].

This article was previously posted to the medRxiv preprint server on February 23, 2024.

## Case presentation

A 19-year-old female with a history of a rare diagnosis of RSTS was admitted to the hospital in March 2021 with a large right cheek mass and right leg swelling that had worsened for two months with associated night sweats. She was intellectually impaired, so her history was taken from her mother, the primary caregiver. No family history of lymphomas was reported.

**Figure 1:**
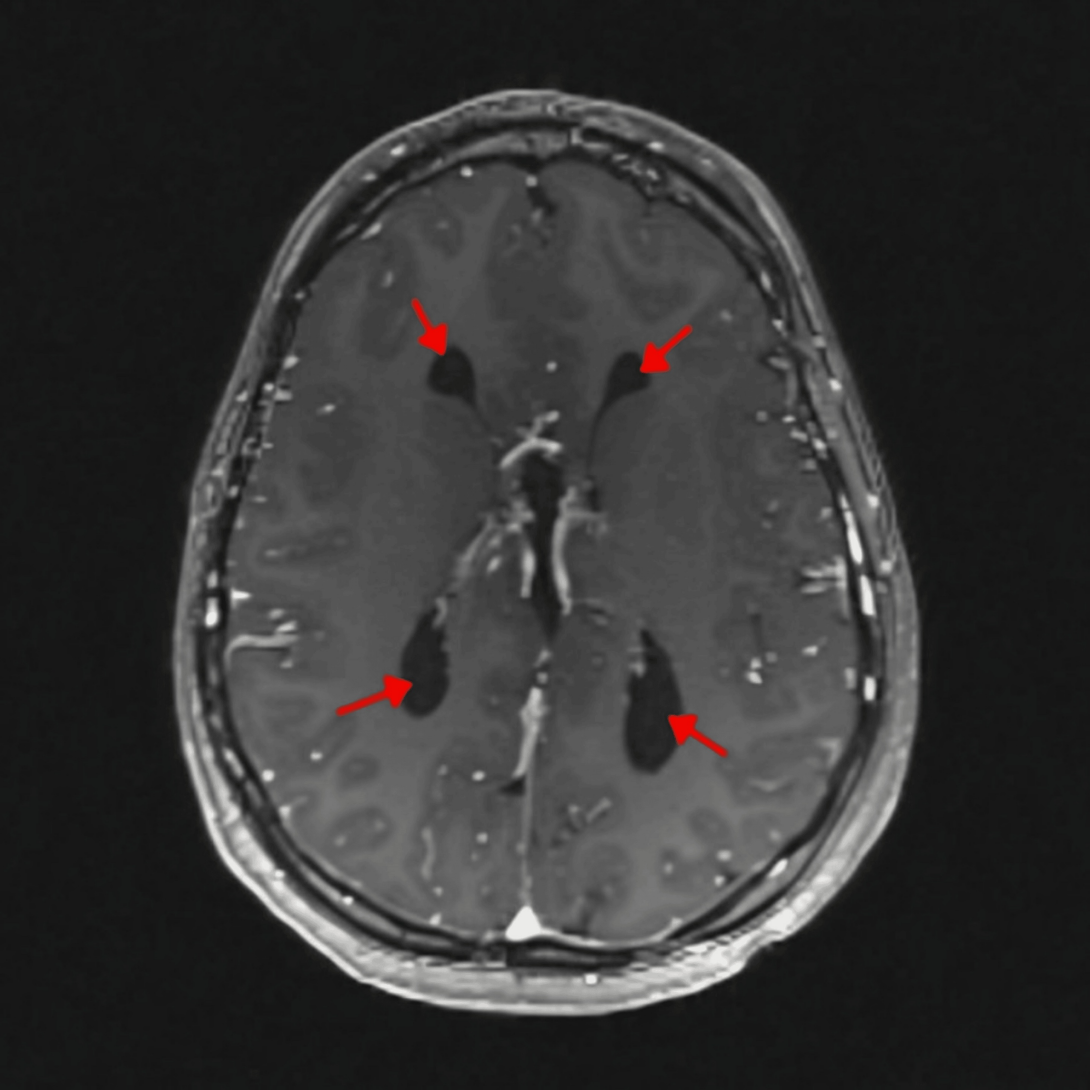
MRI brain shows widely separated lateral ventricles parallel to each other (red arrows), a typical feature of agenesis of the corpus callosum.

**Figure 2:**
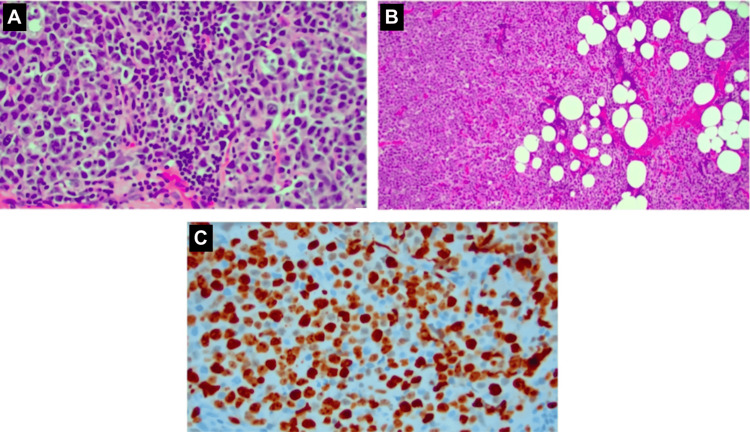
(A) H&E 40× image of a lymph node biopsy showing diffuse large B-cell lymphoma cells. (B) H&E 10× image of a lymph node showing sheets of diffuse large B cells. (C) 40× image of Ki-67 on the lymph node biopsy indicating high proliferation.

On physical examination, she was significant for her short stature, narrow head with close-set eyes, mild obesity, distinct facial features, and broad thumbs, and the right lower extremity has thickened skin along with right thigh swelling. Lab abnormalities included elevated bilirubin, lactate dehydrogenase (LDH), and uric acid levels (Table 1). A CT scan of the abdomen and pelvis revealed a 9.5 x 10.4 cm soft tissue mass in the pelvis that was displacing the bladder and uterus, encasing the right iliac vessels, and attaching to the right pelvic sidewall. There were several large soft tissue masses in the right inguinal region and retroperitoneal lymph nodes, as well as multiple compartmental soft tissue masses in the right pelvis extending into the inguinal canal and the anterior thigh. CT chest showed small pleural effusions on both sides. CT neck showed enhancing soft tissue mass in the right parotid gland with enlarged anterior cervical chain lymph nodes. The brain MRI showed the congenital absence of the corpus callosum (Figure 1). The right groin lymph node biopsy showed diffuse large-cell lymphoma of germinal center origin, which is Ki-67 95% (Figure 2), BCL 6, and MYC immunostaining positive but CD 30, t(8,14), and Epstein-Barr virus (EBV) PCR negative. A bone marrow biopsy and CSF analysis showed no evidence of lymphoma. Next-generation sequencing analysis is a cancer genomic profiling test performed using the FoundationOne CDx kit (Foundation Medicine, Inc., Boston, MA, USA), which sequences DNA regions of interest in a tumor sample. Analysis on lymph node biopsy showed tumor mutation burden (TMB) low intermediate at 6, microsatellite stability (MSS), mutations in*BRAF K601N*,*CREBBP R1319*,*FOX01 MV*, and* TNFRSF14* alteration. Liquid biopsy (FoundationOne) indicated* *the*CREBBP R1319* mutation.

**Table 1:** Laboratory test results The patient's abnormal lab values show high bilirubin, LDH, and uric acid levels, with a Ki-67 of 95% and an intermediate TMB. LDH: lactate dehydrogenase; TMB: tumor mutation burden

Parameters	Patient's Values	Normal Range	Unit
Bilirubin	1.9	0.2-1.2	mg/dL
LDH	718	117-278	U/L
Uric acid	8.7	2.6-6.0	mg/dL
Ki-67	95	0-100	%
TMB	6	Low: ≤ 5, Intermediate: 6-20, High: 21-50, Very High: 51+	mut/Mb

**Figure 3:**
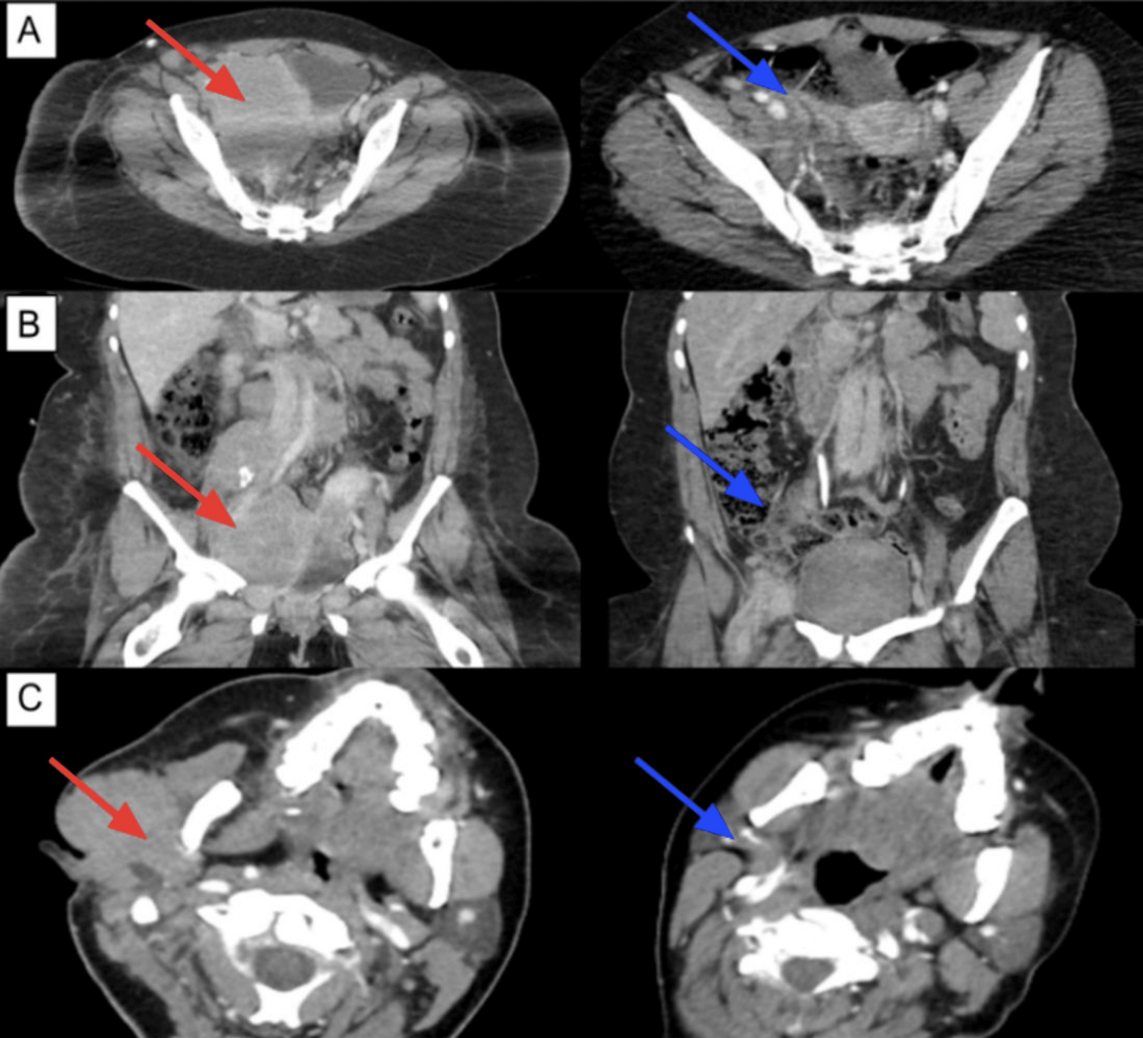
(A) Axial CT images of pelvic lymphoma before (red arrow) and after (blue arrow) two cycles of treatment with R-EPOCH. (B) Coronal CT images of paraaortic and aortocaval lymphoma before (red arrow) and after (blue arrow) treatment. (C) Axial CT images of cheek lymphoma before (red arrow) and after (blue arrow) treatment.

The patient was started on treatment with chemotherapy with Rituxan with EPOCH (R-EPOCH) chemotherapy. R-EPOCH was chosen due to high-risk features and suspicion of double HIT lymphoma while we were awaiting C-MYC analysis. By cycle two of chemotherapy, there was an 80% response rate. The patient completed six cycles of R-EPOCH, and the post-treatment PET scan continued to show no activity (Figure 3). She is now two years out from treatment with no activity on PET and residual lymph nodes on CT.

## Discussion

RSTS is a rare genetic disorder primarily attributed to mutations in the *CREBBP* gene, which plays a pivotal role in the syndrome's pathogenesis. Notably, individuals with RSTS are predisposed to malignancies due to the *CREBBP* genetic aberration. In this exceptional case, we have delved into the intricate relationship between RSTS and its association with a high-risk variant of diffuse large B-cell lymphoma (DLBCL), further shedding light on targeted treatment possibilities. It is known that *CREBBP*-encoded transcriptional co-regulatory protein interacts with other transcription factors and controls gene expression. As seen in this case, alteration in the *CREBBP* gene can disrupt its function and expression.

We are reporting the patient's excellent response to treatment despite classic unfavorable prognostic markers, such as high Ki-67 and high LDH levels, and unfavorable genomic aberrations, such as the presence of *CREBBP*, *BRAF K601N*, *CREBBP R1319*, *FOX01 MV*, and *TNFRSF14* alterations. The observed complete remission on PET scans following EPOCH chemotherapy encourages the consideration of escalated treatment strategies such as R-EPOCH in cases involving *CREBBP* gene mutations. Moreover, recognizing the presence of both somatic and germline *CREBBP* mutations adds weight to the potential pathogenic role of these alterations in the context of this rare syndrome. Evidence of the emerging therapeutic role of *CREBBP/EP300* inhibitors in malignancies has been noted in previous articles, indicating potential tools for clinical intervention [11].

Intriguingly, the findings from this case have broader implications for other sporadic DLBCLs. The presence of *CREBBP* or *BRAF K601N* gene alterations, as observed in our case, which responded well to intensified therapy with the R-EPOCH regimen, supports the rationale for escalated treatment strategies across certain molecular subsets like *CREBBP* in DLBCLs. This implies that a more personalized therapeutic approach, tailored to the genetic landscape of the malignancy, might be a pivotal factor in achieving comprehensive remission.

It is important to acknowledge the limitations of this study. Although R-CHOP is considered the standard treatment, R-EPOCH was given due to the high Ki-67 and suspicion of double HIT lymphoma. Whether the patient would have gotten a similar complete response if R-CHOP chemo had been initiated is unknown.

## Conclusions

This case provides genomic sequencing insights into a rare case of RSTS-associated high-risk DLBCL and acts as a prototype for exploring innovative treatment approaches. The distinct response observed in this case warrants further investigation into the potential benefits of intensified therapies in DLBCL patients harboring specific genetic alterations such as *CREBBP* and *BRAF*. Ultimately, this case encourages continued research into the interplay between genetic mutations, syndrome-related predispositions, and effective therapeutic strategies for patients with rare syndromes and associated malignancies.
